# Supplementary material for: Abundance and Compositions of B-Vitamin-Producing Microbes in the Mammalian Gut Vary Based on Feeding Strategies
Source: mSystems. 2021 Aug 31;6(4):10.1128/msystems.00313-21. doi: 10.1128/msystems.00313-21 (PMC12338137; doi:10.1128/msystems.00313-21)
Supplement: FIG S1 [file msystems.00313-21-sf001.pdf]

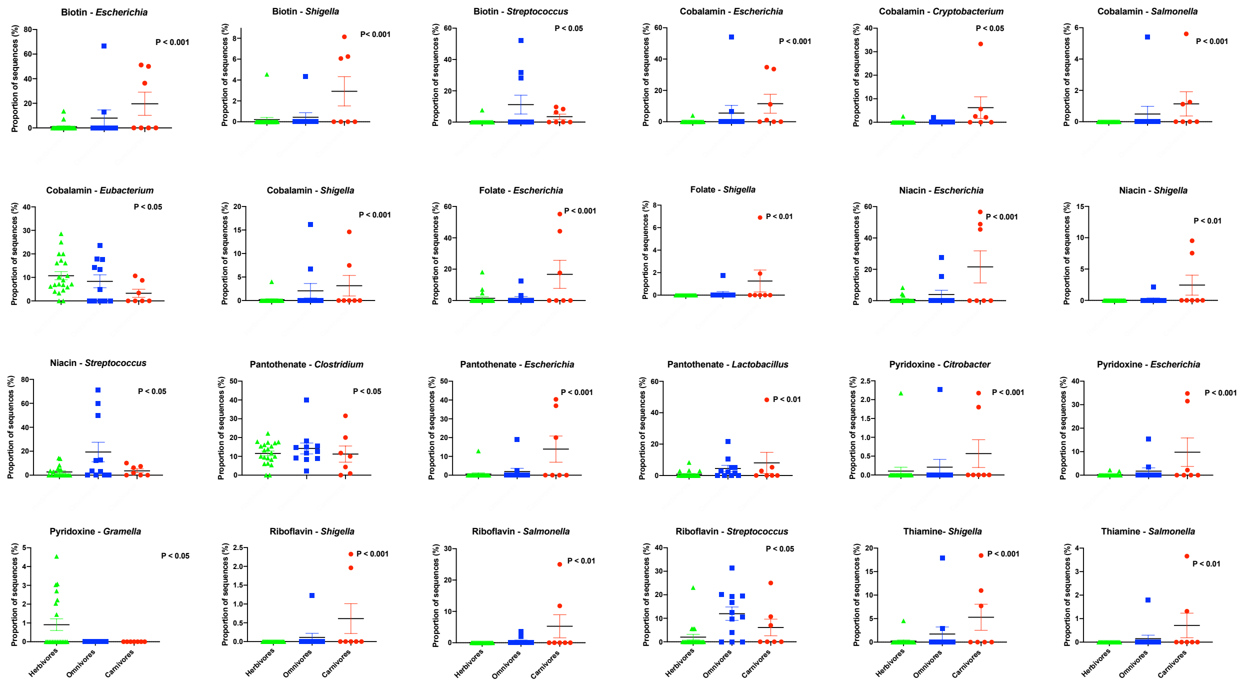

Supplementary Fig.1: Genus identified to vary between the three treatment groups based on feeding strategy are shown in the taxon plots. P values are determined by Kruskal-Wallis one-way analysis of variance (ANOVA) with *Games-Howell* 's post hoc test.
