# Supplementary material for: Abundance and Compositions of B-Vitamin-Producing Microbes in the Mammalian Gut Vary Based on Feeding Strategies
Source: mSystems. 2021 Aug 31;6(4):10.1128/msystems.00313-21. doi: 10.1128/msystems.00313-21 (PMC12338137; doi:10.1128/msystems.00313-21)
Supplement: FIG S2 [file msystems.00313-21-sf002.pdf]

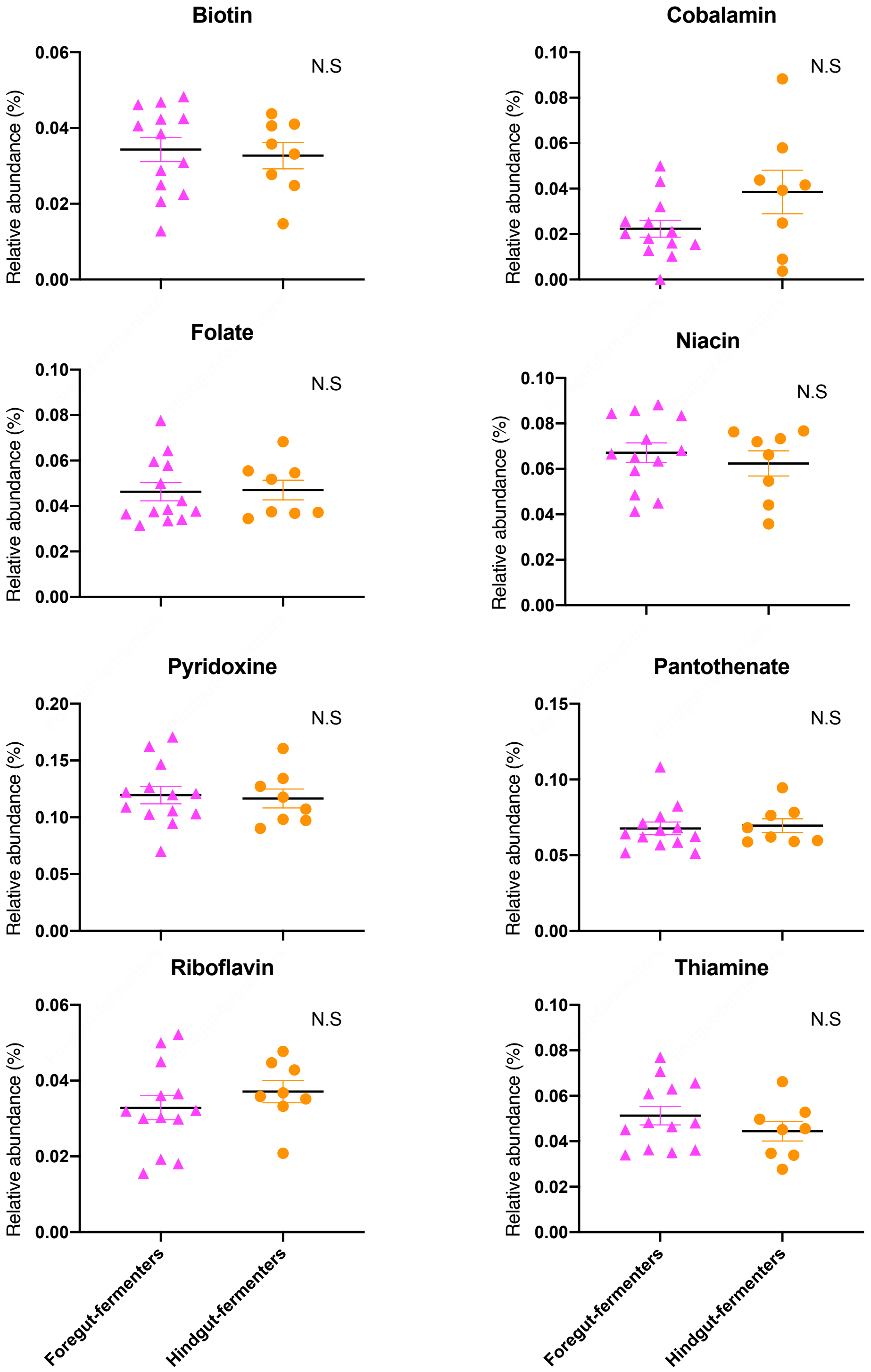

Supplementary Fig. 2: The relative abundance of genes associated with B-vitamin synthesis in hindgut and foregut fermenting herbivores. We did not find a significant difference in gene abundance between the two groups when tested with t-test.
