# Supplementary material for: Abundance and Compositions of B-Vitamin-Producing Microbes in the Mammalian Gut Vary Based on Feeding Strategies
Source: mSystems. 2021 Aug 31;6(4):10.1128/msystems.00313-21. doi: 10.1128/msystems.00313-21 (PMC12338137; doi:10.1128/msystems.00313-21)
Supplement: FIG S3 [file msystems.00313-21-sf003.pdf]

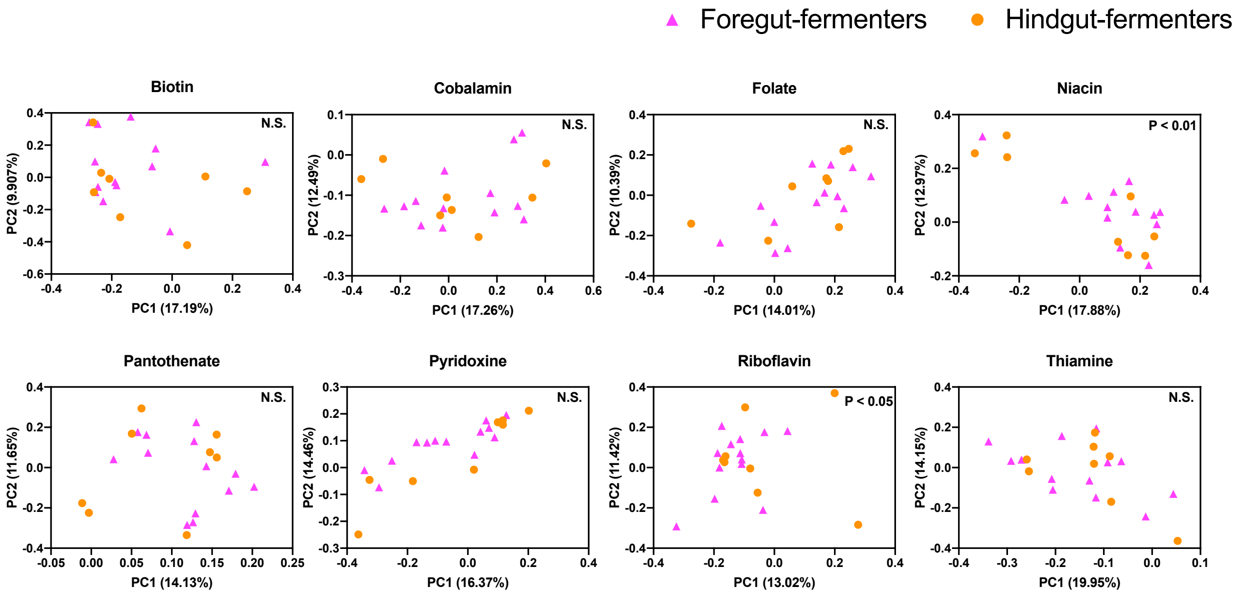

Supplementary Fig 3: The structure of B-vitamin synthesizing microbes identified in hindgut and foregut fermenting herbivores. The distance matrices were generated from Bray-Curtis dissimilarity metric for bacteria involved in B-vitamin synthesis. Niacin and riboflavin were the only vitamins to have a significantly different microbial structures ($p < 0.05$), as it was assessed by PERMANOVA of the dissimilarity of the Bray-Curtis dissimilarities.
